# The impact of a changing winter climate on the hatch phenology of one of North America’s largest Atlantic salmon populations

**DOI:** 10.1093/conphys/coz015

**Published:** 2019-05-15

**Authors:** Anna C Rooke, Brittany Palm-Flawd, Craig F Purchase

**Affiliations:** Biology Department, Memorial University of Newfoundland, St. John's, Newfoundland and Labrador, Canada

**Keywords:** Climate change, emergence, incubation, phenology, *Salmo salar*, salmonid

## Abstract

In northern temperate regions, some of the most dramatic effects of climate change are expected during the winter. Understanding how changing winter climates influence the seasonal timing of key life events is critical for implementing effective conservation strategies, especially for poikilotherms, whose physiology and development are particularly sensitive to changes in thermal environment. Four mathematical models are available to predict the timing of hatch and emergence in Atlantic salmon (*Salmo salar*); however, such models are only useful if the effect of temperature is both repeatable within and among maternal families, and predictable across variable temperature regimes. Using a split-brood experiment, we found the timing of hatch to be repeatable and predictable in Atlantic salmon from the Exploits River, one of the largest remaining wild populations in North America. Three of the available mathematical models under-estimated the timing of hatch by an average of 21–26 accumulated thermal units (ATU); however, we identified one model that provided reasonable estimates of hatch timing (average under-estimate 7 ATU) under the three incubation temperature regimes we tested. We applied this model to daily water temperature profiles from 2006–18 at four sites within the Exploits River watershed. Across all years and sites, the predicted dates at 50% hatch ranged between 8 March and 23 May, while predicted dates of 50% emergence ranged from 11 May to 13 June. By identifying the seasonal timing of these particularly vulnerable early life stages, this model can aid the implementation of conservation efforts for this ecologically and economically important population.

## Teaser

We identified and tested four developmental models that can be used to predict the timing of hatch in Atlantic salmon from the Exploits River, Newfoundland. This population is one of the largest in North America and can be used as a baseline for monitoring the effects of climate change.

## Introduction

Shifts in reproductive phenology, including the seasonal timing of migration and breeding, are one of the primary phenotypic responses of wild populations to contemporary climate change (Walther *et al.,* 2002; Parmesan and Yohe, 2003; Bradshaw and Holzapfel, 2006; Thackeray *et al.,* 2010). Although most research on reproductive phenology focuses on advancements in spring warming, in northern temperate regions some of the most dramatic effects of climate change will occur during the winter (Christensen *et al.,* 2013). The trend towards shorter, warmer winters in temperate regions will impact the reproductive phenology of any species that incubates or raises young during the winter period. Understanding how changing winter climates influence the seasonal timing of key life events is critical for implementing effective conservation strategies, especially for poikilotherms, whose physiology and development are particularly sensitive to changes in thermal environment.

Many temperate coldwater fishes, such as most salmonids (subfamily, Salmoninae), spawn in the fall and embryos incubate in gravel nests throughout the winter. The timing of hatch, and emergence from the nest in spring, are critical for survival; individuals that emerge early are more likely to establish feeding territory and competitive dominance than those that emerge later; however, if hatchlings emerge too early they may experience high predation and reduced prey availability (Brännäs, 1995; Einum and Fleming, 2000; Skoglund *et al.,* 2011). Spawn timing and incubation temperature are the key factors affecting phenology of hatch, with warmer incubation temperatures resulting in faster physiological development and shorter incubation periods (Peterson *et al.,* 1977; Murray *et al.,* 1990; Berg and Moen, 1999; Geist *et al.,* 2006; Jeuthe *et al.,* 2016). Given the importance of temperature to development, the duration of the incubation period is typically reported in total accumulated thermal units (ATU), an index of time that incorporates total metabolically relevant thermal energy. Embryo development is not directly proportional to temperature; the rate of development per thermal unit is disproportionately faster at temperatures <5°C (Brannon 1987, Brannon *et al.,* 2004). Thus, embryos incubating at cold temperatures require fewer total ATU before hatch than those incubating at warmer temperatures. This developmental compensation is wide-spread in salmonids, and contributes to stabilizing the timing of hatch and emergence in the face of variable thermal environments during incubation (Brannon 1987; Quinn, 2005).

Despite this compensatory effect, the seasonal timing of hatchling emergence is still influenced by inter-annual variability in winter temperatures (Elliott *et al.,* 2000), indicating that changes in winter climate will affect the early life phenology of salmonids independent of the timing of spring warming. Since climate change is expected to have a disproportionate influence on winter climate relative to spring or summer, a better understanding of how thermal conditions during the winter influence the phenology of hatch is needed. Given the importance of phenology to hatchling survival, development of robust empirical models that predict the timing of hatch and emergence would allow fisheries managers to target conservation efforts during this particularly vulnerable period in early life.

Several empirical models have been published to predict the timing of hatch and emergence in salmonids (e.g. Beacham and Murray, 1990; Beer and Anderson, 1997; Elliott and Hurley, 1998; Jensen *et al.,* 2009). Such models typically incorporate the date of spawning, the accumulation of thermal units during incubation and the influence of developmental compensation, to predict the phenology of embryo development. To date, the accuracy of empirical models in predicting the timing of hatch/emergence has been variable (Crisp, 1988; Hendry *et al.,* 1998; Skoglund *et al.,* 2011; Gerson *et al.,* 2016; Sparks *et al.,* 2017; Beer and Steel, 2018; Campbell *et al.,* 2019). This difficulty is in part due to additional factors that influence developmental timing which may not be included in predictive models (e.g. sedimentation and dissolved oxygen: Geist *et al.,* 2006; Franssen *et al.,* 2012, temporal thermal variability: Steel *et al.,* 2012; Beer and Steel, 2018; Penney *et al.,* 2018), as well as the high spatial variability of intra-gravel incubation conditions (Acornley, 1999; Hanrahan, 2007). The utility of species-specific models may also be limited by local thermal adaptation of early life development (Hendry *et al.,* 1998; Purchase and Brown, 2000; Crozier *et al.,* 2008; Jensen *et al.,* 2008; Whitney *et al.,* 2014; Echave *et al.,* 2017). Local adaptation may necessitate population-specific empirical models to make accurate predictions, the development of which would be prohibitive given the complex population structure of salmonids even across small spatial scales.

In order for predictive models to be useful, the effect of temperature on the timing of hatch must be repeatable both among siblings within a family (e.g. male–female pairing), and among families within a population. Maternal effects play a large role in shaping early life phenotype in salmonids (Fleming *et al.,* 2011; Van Leeuwen *et al.,* 2016; Penney *et al.,* 2018; Thorn and Morbey, 2018), and are primarily mediated through egg provisioning: larger eggs produce larger offspring, which can influence survival during this vulnerable life stage (Einum and Fleming, 1999; Thorn and Morbey, 2018). As such, repeatability within and among maternal families specifically, may be important in the precision of model predictions. Useful models must also be robust to variations in the exact pattern of incubation temperature (Penney *et al.,* 2018), which can influence hatch timing independent of the average temperature (Steel *et al.,* 2012). If the timing of hatch is sensitive to how thermal units accumulate during incubation, the precision of a predictive model developed under constant incubation temperatures in the laboratory may be limited when applied to the naturally fluctuating temperatures experienced by eggs incubating in the wild. Fortunately, technological advancements have made gathering detailed temperature data in the wild relatively easy, which helps to minimize the impact of Jensen’s inequality (i.e. performance at the average environmental condition is not equivalent to the average performance across a range of environmental conditions; Ruel and Ayres, 1999) on model predictions by allowing us to apply non-linear development functions over shorter timeframes. Yet, the accuracy of developmental models when applied to such detailed variable thermal regimes is not well tested. This is particularly important given that climate change is expected to increase the variability of thermal conditions in the future.

In this study, we assessed the intra- and inter-maternal family variability of hatch timing in Atlantic salmon (*Salmo salar* Linnaeus, 1758) from the Exploits River, Newfoundland, Canada, under one constant, and two varying thermal incubation regimes. The Exploits River hosts one of the largest remaining wild populations of Atlantic salmon in North America, and as such, is of conservation concern given the global declines in Atlantic salmon abundance in recent decades (COSEWIC, 2010; ICES, 2017). The past and present environmental conditions experienced by the Exploits River population could also serve as a benchmark for future monitoring of changing climates. Considering the importance of phenology for hatchling survival, we predict little intra- and inter-maternal family variability in hatch timing within the population. We compared our observed hatch timing in laboratory conditions with the timing predicted by four previously published models to assess the utility of these models in predicting hatch timing in the Exploits population. Finally, we explore the inter-annual variability in predicted hatch and emergence windows for wild Atlantic salmon from this ecologically, and economically important population.

## Materials and methods

### Study population and gamete collection

We used eggs and semen from Atlantic salmon from the Exploits River, Newfoundland, Canada (48°92'N, 55°66'W). The Exploits River is the largest river (length: 246 km, drainage basin: 11272 km^2^) on the island of Newfoundland, flowing into the Atlantic Ocean. Historically, access to most of the watershed was restricted by natural barriers and hydroelectric facilities; however, improvements in fish-passage technology since the 1960s have dramatically increased access to spawning sites throughout the watershed (Scruton *et al.,* 2008). The Exploits River currently supports one of, if not the largest anadromous Atlantic salmon population in North America, and as such should serve as a key baseline river for monitoring effects of a changing climate. Adults migrate through the lower Exploits River in late June through mid-July (Dempson *et al.,* 2017) and spawning occurs in late October/early November (O’Connell *et al.,* 1983).

In September 2016, wild migrating adult Atlantic salmon were collected from a fish ladder on Grand Falls (48°92'N, 55°66'W), located 22 km upstream from the mouth of the river. Fish were held on site in flow-through tanks until the beginning of November, when gametes were stripped. A total of 18 females (fork length: 567 ± 35 mm (mean ± SD), weight: 1615 ± 279 g) and 18 males (fork length: 544 ± 52 mm, weight: 1335 ± 445 g) were used. Gametes from 3–6 females and 4–5 males were collected on each of four different dates (‘fertilization date’: 1, 4, 8, 11 November 2016), and immediately transported in insulated jars on ice to Memorial University, St. John’s. On arrival (within 10 hours of collection), eggs from each female were divided into 4–5 batches, and each batch was dry fertilized by a single male for ~10 seconds, after which the egg batches were immediately recombined to create a single mixed pool of eggs per female. This fertilization approach helped to maximize genetic diversity among each half-sib maternal family by avoiding sperm competition among males during the first critical seconds of fertilization, and maximizing total fertilization success by then exposing all eggs to sperm from all males. Multiple paternity is common in Atlantic salmon reproduction (Garant *et al.,* 2001; Weir *et al.,* 2010). If paternity has a substantial impact on the timing of hatch, maximizing genetic diversity among half-sib families will increase the within-maternal family variation, and reduce the confounding effect of paternity when comparing among maternal families. Under this design, all females collected on a single fertilization date were fertilized by the same 4–5 males that were collected on the same day. After fertilization, gametes were allowed to water harden for 6–8 hours, and then treated with a disinfecting solution of 10% ovadine, following transfer requirements of Fisheries and Oceans Canada. All procedures performed on live animals were approved by Memorial University’s Animal Care Committee.

### Incubation experiment

**Figure 1:**
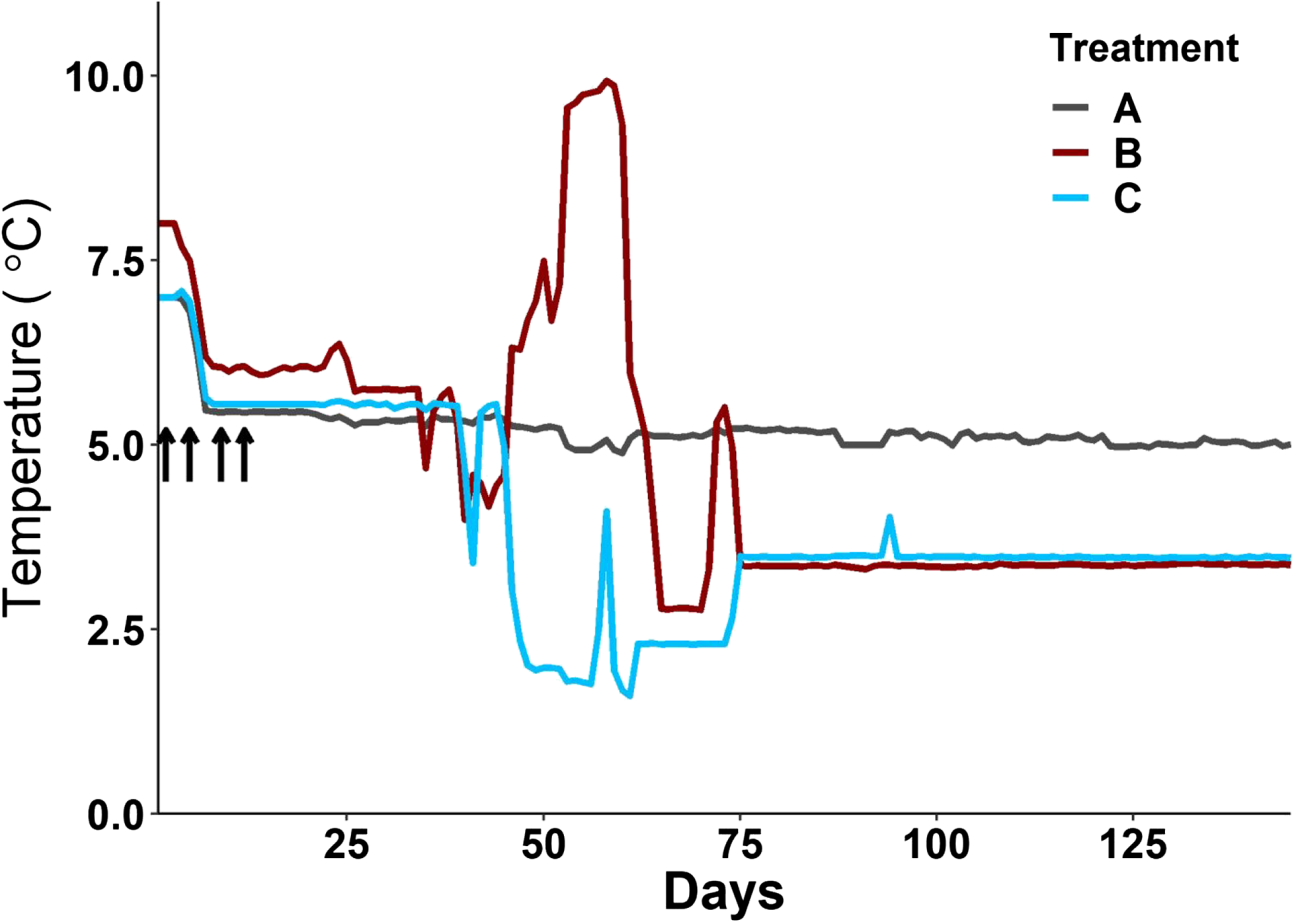
Incubation temperature regimes used in laboratory rearing of Atlantic salmon embryos from the Exploits River. Embryos were incubated at one constant (Treatment A: mean ± SD = 5.3 ± 0.38°C), and two varying (Treatment B: 5.2 ± 1.92°C, and Treatment C: 3.9 ± 1.37°C) thermal regimes. Vertical arrows indicate timing of the four fertilization dates. See Table 1 for average temperatures across the incubation period.

We assessed hatch timing of eggs from each maternal family under three different thermal regimes (Fig. 1; Table 1) to determine if predictive models were robust to variation in how thermal units accumulate over time. Incubation treatment ‘A’ maintained constant water temperature at 5.3 ± 0.38°C throughout incubation. Historical data indicates that the most thermally variable portion of the incubation period in the Exploits River occurs in December, with water temperatures in this large river being relative constant throughout much of the rest of the winter. Climate change is expected to further increase thermal variability in late autumn and early winter, and may result in increased frequency of high flow events due to periodic winter snow melts. Treatments B and C were designed to simulate a large temperature swing in early winter to test how such conditions influence the phenology of incubating embryos. Embryos in incubation treatment ‘B’ were exposed to warmer temperatures (10°C), while embryos in incubation treatment ‘C’ were exposed to cooler temperatures (2°C), for 3–4 weeks in late December and early January. After the period of varying temperatures, both treatments ‘B’ and ‘C’ were held at constant 3.4 ± 0.08°C until hatch (Fig. 1).

**Table 1:** Mean ± SD incubation temperature (°C) from day of fertilization until the last embryo hatched, ATU at 50% hatch (ATU_50_), and days post fertilization at 50% hatch (DPF_50_) for each treatment and fertilization date.

Fertilization date	Treatment A (constant)			Treatment B (warm spike)			Treatment C (cool spike)		
	°C	ATU_50_	DPF_50_	°C	ATU_50_	DPF_50_	°C	ATU_50_	DPF_50_
1 Nov	5.3 ± 0.38	526 ± 3.9	99 ± 0.8	5.2 ± 1.92	534 ± 5.3	100 ± 1.6	3.9 ± 1.40	492 ± 2.9	124 ± 0.8
4 Nov	5.3 ± 0.24	526 ± 9.2	100 ± 1.8	5.2 ± 1.90	531 ± 3.0	103 ± 0.9	3.9 ± 1.33	485 ± 6.9	125 ± 2.0
8 Nov	5.2 ± 0.15	534 ± 2.7	102 ± 0.5	5.1 ± 1.91	515 ± 2.2	102 ± 0.9	3.8 ± 1.25	471 ± 7.0	124 ± 2.0
11 Nov	5.2 ± 0.15	521 ± 1.5	99 ± 0.3	5.2 ± 1.94	501 ± 2.2	101 ± 0.7	3.7 ± 1.24	476 ± 3.8	127 ± 1.1
Treatment total	5.3 ± 0.38	527 ± 7.1	100 ± 1.7	5.2 ± 1.92	521 ± 12.9	102 ± 1.5	3.9 ± 1.37	481 ± 9.6	125 ± 2.0

Embryos were incubated in vertical re-circulating salmonid incubators (Marisource, www.marisource.com) located in a dark, temperature controlled room (5°C). Temperature in the variable incubation treatments were controlled by heaters and chillers, and recorded every hour (HOBO Water Temp Pro v2 data loggers, www.onsetcomp.com). Incubators were filled with degassed tap water, fitted with canister water filters with three stages of filtration, UV filters and received constant aeration to maintain 100% oxygen saturation. Water in the incubators was partially exchanged every other day in order to maintain high-water quality parameters (pH: 7.6 ± 0.5, ammonia: 0.5 ± 0.2 ppm).

In a split-brood design, viable (non-white) embryos from each maternal family were randomly selected 10–16 hours after fertilization and 50 embryos were incubated in 50 mm (diameter) by 80 mm (height) dark plastic polyvinyl chloride (PVC) tubes with mesh covers on either end. Four to six replicate incubation tubes from each maternal family were reared in treatment ‘A’ (i.e. constant 5.3°C); however, due to logistical constraints, only one incubation tube per maternal family was reared under each of the varying temperature treatments (i.e. treatments ‘B’ and ‘C’). Repeatability analysis of replicate incubation tubes in the constant temperature treatment (see Results) indicates that data from a single incubation tube is a good representation of an individual maternal family’s hatch timing. Replicate incubation tubes and/or maternal families were distributed across all four trays in an incubator, and there was no temperature gradient from the top to bottom tray. Embryos were inspected twice each week and all dead (white) eggs were removed to reduce the risk of fungal infection. Once embryos reached the eyed stage, they were checked daily and the number of hatched and unhatched were recorded.

### Data analysis

Embryos from 18 different maternal families were incubated; however, hatch success was very low in one maternal family (0–14%) and this family was removed prior to analysis. Hatch success among the remaining maternal families varied widely (34–78%), but was relatively constant within a family even when reared under different incubation regimes (see Supplementary Figure A). To incorporate the impact of temperature on development, we report the timing of hatch in ATU (the sum of average daily water temperature) post fertilization. The timing at 50%, and 90% hatch was estimated with logistic regression fitted to hatch data for each incubation tube. All data analyses were completed in R 3.4.3 (R Development Core Team, 2017).

We assessed intra- and inter-maternal family repeatability of hatch timing using replicate incubation tubes held under the constant incubation treatment. As a measure of repeatability within maternal families, we report the intra-class correlation coefficient (‘ICC*’* package; Wolak *et al.,* 2012); an ICC close to 1 indicates that most of the variability is explained by the variation among maternal families, and therefore the trait is considered highly repeatable within maternal families. To assess repeatability among families, we used a mixed model approach with fertilization date included as a fixed effect, and individual maternal family as a random intercept:

$$ Hatch\ Timing=\alpha +{\beta}_1 Fertilization\ Date+{b}_{\mathrm{maternal}\ \mathrm{family}}+\epsilon, $$

where α is the global mean hatch timing, ${\beta}_1$ is the coefficient associated with each of the four fertilization dates and $\epsilon$ is the random error term. Fertilization date was included to account for small differences in the thermal regimes experienced by embryos that were fertilized on the four different dates (Fig. 1; Table 1). In this model, a significant maternal family effect indicates differences in hatch timing among maternal families fertilized on the same day.

The effect of incubation temperature regime on hatch timing was also assessed using a mixed model approach. Incubation treatment (‘A’, ‘B’ and ‘C’), fertilization date, and the interaction between treatment and date were included as fixed effects, and individual maternal family was included as a random intercept. Only a single incubation tube per maternal family was reared in treatments ‘B’ and ‘C’, therefore we averaged the replicate measures in treatment ‘A’ to obtain a single measure per maternal family used in this model. Since embryos from all maternal families were incubated in all three treatments only once, a significant maternal family intercept effect in this model indicates that hatch timing in all maternal families responded to different thermal regimes in a similar way (i.e. a maternal family that required relatively few ATU at hatch in one treatment also tended to require relatively few ATU in another treatment).

Mixed models were analysed using the ‘lme4’ package (Bates *et al.,* 2014) and the Satterthwaite approximation (‘lmerTest’, Kuznetsova *et al.,* 2015), with the significance of the random effect assessed using a likelihood ratio test. Summary statistics of all mixed model analyses are available in Supplementary Material B. Residuals of statistical models met the assumptions of normality (Shapiro–Wilk Test) and homogeneity of variance (Levene Test) at a significance level of α = 0.05 without transformation. We completed all analyses on ATU at 50% and 90% hatch because the predictive models available in the literature (see below) use both these measures of hatch timing. However, since the results were very similar, here we only present the analysis on ATU at 50% hatch as it is the most commonly used measure of hatch timing. Complete results of ATU at 90% hatch are available in the Supplementary Material C.

### Evaluating published model predictions

To determine if available predictive models can provide reasonable estimates of the timing of hatch for the Exploits River population, we compared our observed ATU at hatch with ATU predicted by the four hatch/emergence models for Atlantic salmon that we could find in the literature (see below). All four models use non-linear thermal growth relationships to predict incremental development during discrete time steps (e.g. days or weeks). Hatch and/or emergence is predicted to occur once a specific sum of development is accumulated. Since embryos fertilized on different dates in our laboratory experiment experienced slightly different thermal regimes (Table 1; Fig. 1), we compared the average observed ATU at hatch with the ATU predicted by each model, for every fertilization date and incubation treatment. For each of the four predictive models, we used a paired *t*-test (data paired by fertilization date and incubation treatment, *n* = 12) to determine whether model predictions were significantly different from the observed values.

The Crisp (1981) model uses daily percent development relative to daily average temperature (*T*_avg_) to predict days from fertilization until 50% hatch.

\begin{align*} \log \left( days\ to\ hatch\right)=&\,-2.6562\log \left({T}_{\mathrm{avg}}+11.0\right)\\ &+\log \left(-11.0\right){\hskip48pt}\textrm{Crisp model} \end{align*}

This model was developed and parameterized using previously published data on hatch timing from British (River Kent—Carrick, 1979), Norwegian (Gunnes, 1979) and Canadian (Miramichi River, New Brunswick—Peterson *et al.,* 1977) Atlantic salmon populations reared in the laboratory between 2.4–13°C.

The Gorodilov (1996) model describes the cumulative thermal energy required to reach over 100 morphologically discrete developmental states during early Atlantic salmon development. It is based only on daily average temperature and the accumulation of T_s_ (a measure of relative age in minutes, representing total thermal energy required to develop one somite pair).

\begin{align*} \log \left(T_s\right)=&\ 3.0984-0.0967{T}_{avg}+0.00207{T_{avg}}^2 \\&{\hskip130pt}\textrm{Gorodilov model} \end{align*}

A specific developmental state is reached when a designated number of T_s_ is accumulated (see Table 1 in Gorodilov, 1996). We used this model to predict the number of days until ‘peak hatch’ (i.e. 50% hatch: 315 T_s_) and emergence (450 T_s_). The Gorodilov model was developed using Atlantic salmon from hatcheries located on the Neva, Narova and Salaca rivers (draining into the Baltic Sea) and the Kola river (draining into the Barents Sea), reared under 13 constant temperature regimes between 0.1–11.0°C.

The WinSIRP model (Jensen *et al.,* 2009) is designed to assist fish culturists and biologists to predict developmental timing in several species of salmonids. This computer model predicts ATU at 50% hatch using weekly average temperatures (T_wk_)

$$ days\ to\ hatch= \frac{11248}{{\left({T}_{wk}+5.3944\right)}^{2.0198}}{\hskip30pt}\textrm{WinSIRP model} $$

and incorporates the influence of dissolved oxygen, pH, flow rate and waste production on development, which are particularly important when rearing embryos at high densities. The WinSIRP model was parameterized for Atlantic salmon from broodstocks maintained at the Glacier Bay site in Jervis Inlet (British Columbia, Canada), originating from the MOWI (Norwegian) and Cascade (Canadian—Gaspé, Québec) aquaculture stocks, and incubated under constant temperature regimes between 4–14°C.

The Kane model (Kane, 1988) uses daily percent development to estimate ATU at 90% hatch and emergence, and relies only on daily average temperatures.

$$ \ln \left( days\ to\ hatch\right)=5.483{e}^{-0.0347{T}_{avg}} {\hskip40pt}\textrm{Kane model}$$

This model was developed using Atlantic salmon from the Penobscot River (Maine, U.S.) incubated under 13 seasonally varying thermal profiles.

The Gorodilov and Kane models have been used primarily to assess the developmental stage of Atlantic salmon during rearing (Letcher *et al.,* 2004; Moen *et al.,* 2010; Bloomer *et al.,* 2016; Kahar *et al.,* 2016). Both the Crisp and WinSIRP models have been used to predict hatch and emergence dates in hatchery and wild salmon populations with varying degrees of success (Atlantic salmon: Jensen *et al.,* 1991, Skoglund *et al.,* 2011, Hedger *et al.,* 2013; Chinook *Oncohynchus tshawytscha* Walbaum 1792: Unwin *et al.,* 2000; Gerson *et al.,* 2016; Sockeye *Oncorhynchus nerka* Walbaum, 1792: Hendry *et al.,* 1998).

### Predicting hatch and emergence in the wild Exploits River population

The Gorodilov model produced the most accurate predictions of hatch timing in our experiment (see Results), and therefore we used the Gorodilov model to predict hatch and emergence in the wild Exploits River population from 2006–18. We predicted hatch and emergence of embryos fertilized on 4 October, and on 7 November, which corresponds to an assumed two week spawning window (O’Connell *et al.,* 1983). Embryo and alevin (term used to describe hatched salmonids before they emerge from the gravel) development were predicted using daily average water temperatures available from temperature loggers (Water Resources Department, Government of Newfoundland and Labrador, https://www.mae.gov.nl.ca/wrmd/ADRS/v6/Graphs_List.asp) located in free-flowing water at four sites within the Exploits River system: East Pond Brook (48°40'55"N, 56°30'36"W), Gills Pond Brook (48°38'26"N, 56°31'40"W), Great Rattling Brook (48°49'36"N, 55°31'43"W), and the main Exploits River below the Noel Paul’s Brook tributary (48°50′39”N, 56°16′10”W).

## Results

### Intra- and inter-maternal family repeatability

**Figure 2:**
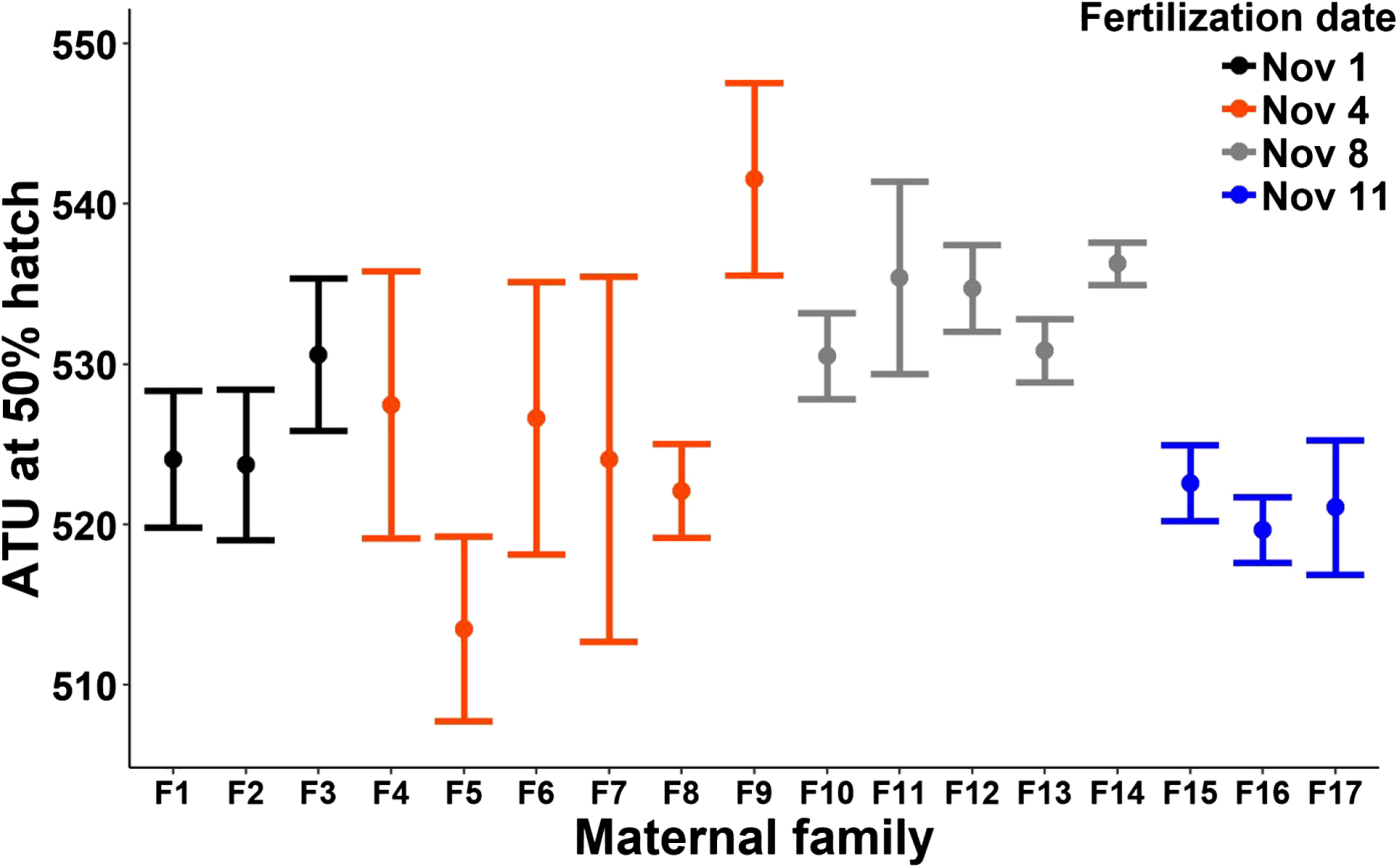
Intra- and inter-maternal family variability in ATU post fertilization at 50% hatch for Atlantic salmon embryos from the Exploits River incubated at constant ~5.3°C (Treatment A). Points show average ± SD of 4–6 replicate incubation tubes per maternal family. Colours depict embryos fertilized on different dates in November 2016.

When incubated at a constant 5.3°C, the ATU required for 50% hatch was considered moderately repeatable among replicate incubation tubes within maternal families (ICC = 0.61; 95% CI: 0.39–0.80). Although hatch timing was significantly different among maternal families (χ^2^_1_ = 21.8, *P* < 0.0001; Fig. 2), the maximum difference between two families (28 ATU, F5 vs. F9, Fig. 2) was small relative to the average total ATU at 50% hatch across all maternal families (527 ATU). Thus, although statistically significant, the among-family variability in hatch timing represented only 5.3% of the average total incubation period. Similarly, the date of fertilization had a significant (F_3,17.5_ = 3.67, *P* = 0.03; Table 1) but modest influence on the timing of hatch: maternal families fertilized on 11 November required significantly fewer ATU to hatch than those fertilized 3 days earlier on 8 November (Tukey: *P* = 0.009); however, the difference was only 12 ATU, which represents only 2.3% of the total average incubation period. Considered together, intra- and inter-maternal family variability in hatch timing was small relative to the total duration of incubation, indicating that the timing of hatch was similar among maternal families.

### Effect of thermal regime on timing of hatch

**Figure 3:**
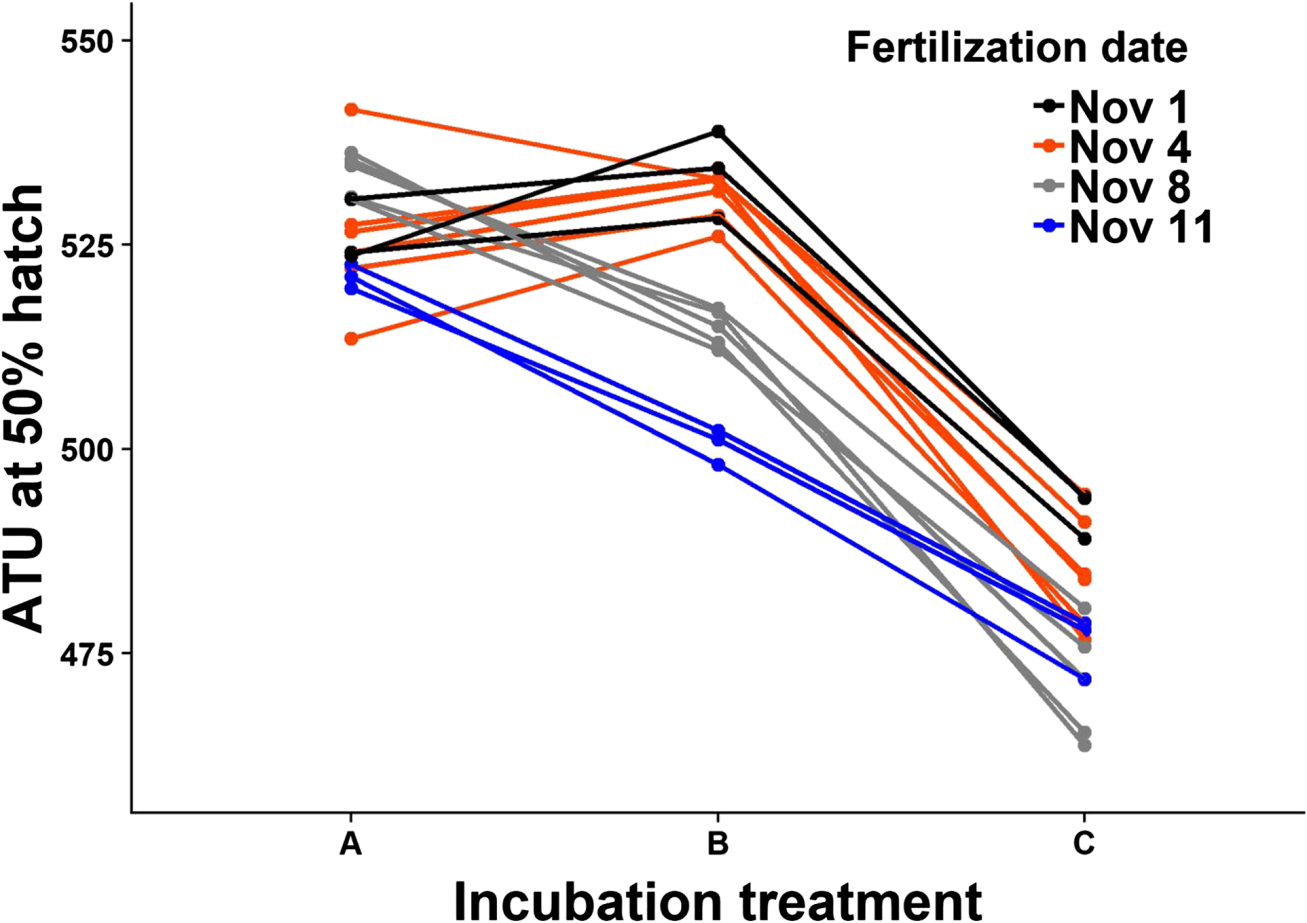
ATU post fertilization at 50% hatch for Atlantic salmon from the Exploits River exposed to different incubation temperatures. Each line represents embryos from a single maternal family incubated at constant (Treatment A: mean ± SD = 5.3 ± 0.38°C), and varying (Treatment B: 5.2 ± 1.92°C—warm spike, Treatment C: 3.9 ± 1.37°C—cold spike) thermal conditions. Colours depict embryos fertilized on different dates in November 2016.

A significant interaction between incubation treatment and date of fertilization (F_6,26_ = 19.1, *P* < 0.0001) precluded a statistical evaluation of the effect of temperature on hatch timing; however, the response to temperature is clear from Fig. 3: embryos incubated at constant 5.3°C, and those exposed to a warm spike during the incubation period, had similar average ATU at hatch, while those exposed to a cold spike during the incubation period required fewer ATU to hatch. Given the average temperature experienced during incubation (Table 1), this pattern is consistent with the effects of compensatory development, where embryos reared at cooler temperatures required fewer ATU at hatch. Although the timing of hatch differed a little among maternal families, the effect of temperature was consistent across maternal families (i.e. a maternal family that required relatively few ATU at hatch in one incubation treatment also tended to require relatively few ATU at hatch in another treatment, χ^2^_1_ = 8.04, *P* < 0.005; Fig. 3).

The date of fertilization influenced the timing of hatch when embryos were exposed to varying temperatures (Fig. 3; Table 1). When exposed to warm temperatures, and to a lesser degree when exposed to cold temperatures, maternal families fertilized on 8 and 11 November tended to require fewer ATU at 50% hatch, compared to maternal families fertilized on 1 or 4 November. However, the largest difference in ATU between fertilization dates (33 ATU, 1 November vs. 11 November in Treatment B, Fig. 3) was small compared with the average total ATU at hatch across all families in the treatment (521 ATU). Thus, the effect of fertilization date on hatch timing was relatively small, representing at most 6.3% of the average duration of the entire incubation period within a treatment.

### Evaluating published model predictions

**Figure 4:**
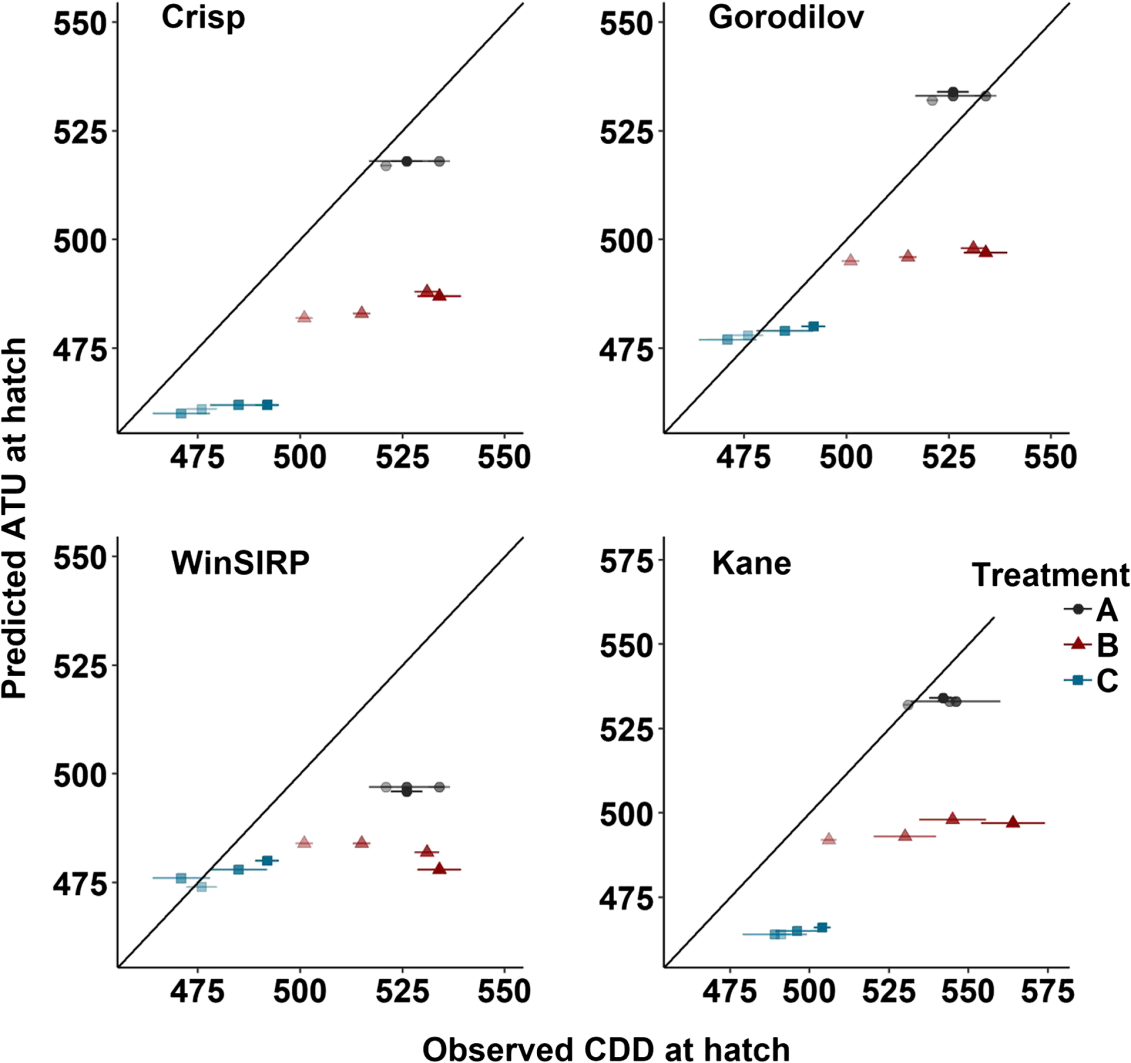
Observed vs. predicted ATU at hatch for Exploits River Atlantic salmon embryos incubated in constant ~5.3°C (Treatment A), and varying (Treatment B—warm spike; Treatment C—cold spike) thermal conditions (symbol colour). Each point represents the mean (± SD) for all maternal families fertilized on the same day. Shading reflects date of fertilization (dark to light: 1 Nov, 4 Nov, 8 Nov, 11 Nov). Solid line = 1:1 line. The Crisp (1981), Gorodilov (1996) and WinSIRP (Jensen *et al.,* 2009) models predict ATU at 50% hatch, while the Kane model (Kane 1988) predicts ATU at 90% hatch.

The Crisp (t_11_ = −5.28, *P* = 0.0003), WinSIRP (t_11_ = −4.53, *P* = 0.0008) and Kane (t_11_ = −4.80, *P* = 0.0006) models significantly underestimated the ATU required at hatch (average difference—Crisp: −21 ATU; WinSIRP = −24 ATU; Kane = −26 ATU). Under the temperatures experienced during hatch in our experiment, these models underestimated the date of hatch by 4–8 days. The predictions from the Gorodilov model were not significantly different from observed values (t_11_ = −1.45, *P* = 0.17; Fig. 4). The Gorodilov model predicted hatch timing in our experiment within 2 days of the observed average. We conclude that the Gorodilov model produces reasonably accurate predictions of timing of hatch in the Exploits River Atlantic salmon population.

### Predicted hatch and emergence timing of wild Exploits River population

**Figure 5:**
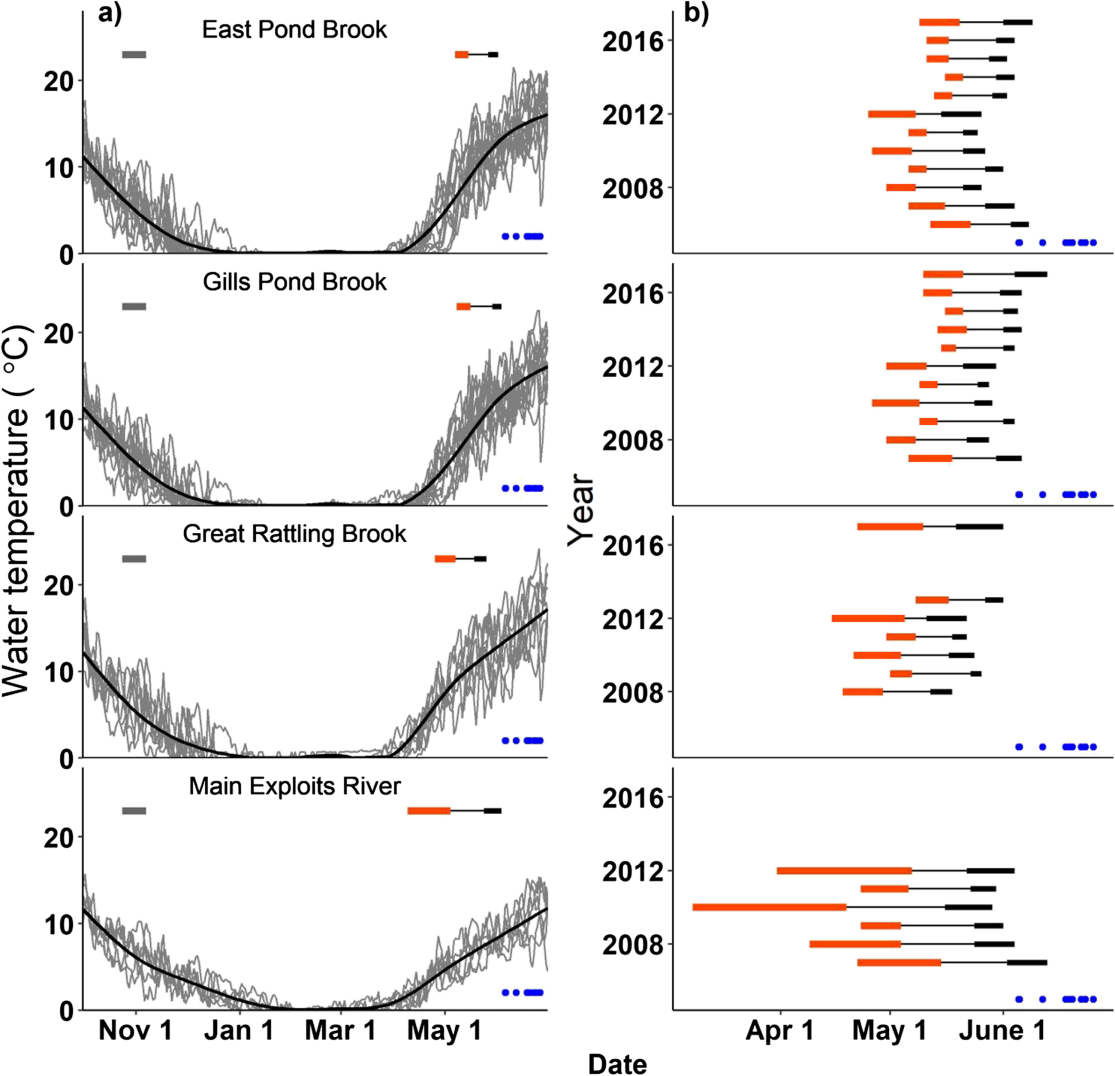
**a**) Water temperature (°C) at four sites in the Exploits River (grey lines = individual years (2006–18), black = average). The assumed spawning period (24 Oct–7 Nov—grey horizontal bar) and average predicted hatch (horizontal orange bar) and emergence (thick horizontal black bar) periods are shown. **b**) Yearly hatch (orange bar) and emergence (thick black bar) periods predicted by the Gorodilov model (see Results). Blue circles show date of 50% emergence observed by O’Connell *et al.* (1983) in an artificial spawning stream in Noel Paul’s Brook from 1970–80.

Based on in-stream temperatures, the predicted timing of hatch and emergence in the wild (using the Gorodilov model: see above) varied among sites, and among years in association with the local thermal conditions of the Exploits River (Fig. 5). Among years, the predicted dates at 50% hatch ranged between 8 March and 23 May (76-day window), while predicted dates of emergence ranged from 11 May to 13 June (33-day window). The average predicted period of emergence within a year (6.8 ± 3.0 days) was shorter than the average predicted period of hatch (11.7 ± 8.5 days). The only site located within the main stem Exploits River (below Noel Paul’s Brook tributary and downstream of a large lake) had warmer mid-winter temperatures and slower spring warming compared with the other three sites from smaller tributaries. This difference in thermal regime resulted in this site having the most protracted predicted hatch and emergence periods (Fig. 5). At all sites, the Gorodilov model predicted emergence to occur earlier in spring than has been previously observed near a hatchery in Noel Paul’s Brook (Fig. 5; see Discussion).

## Discussion

Empirical models used to predict hatch timing rely on the non-linear relationship between incubation temperature and embryo development. This relationship can vary among populations (Murray *et al.,* 1990; Solberg *et al.,* 2014; Whitney *et al.,* 2014; Fuhrman *et al.,* 2018), and among families within a population (Konecki *et al.,* 1995; Solberg *et al.,* 2014; Fuhrman *et al.,* 2018). In addition to the role of compensatory development in stabilizing early life phenology (Brannon *et al.,* 2004; Quinn, 2005), the specific pattern in which thermal units accumulate over time can also influence the timing of hatch independent of the average temperature (Steel *et al.,* 2012; Beer and Steel, 2018). Our laboratory experiment indicates that the ATU required to hatch in Exploits River Atlantic salmon is repeatable within maternal families, and that inter-family variability was relatively small, representing <8% of the average total duration of the incubation period. The effect of temperature on hatch timing was relatively consistent, with maternal families tending to respond to different incubation temperatures in a similar way. This consistency indicates that an accurately parameterized empirical model can predict hatch timing in this population relatively precisely.

We assessed whether previously published models for Atlantic salmon can accurately predict hatch timing in the Exploits River population incubated in laboratory conditions. Under the temperature regimes we tested, the Crisp, WinSIRP and Kane models tended to underestimate the ATU required at hatch. The difference between observed and estimated timing (up to 65 ATU) is well within the maximum inter-population variability documented in other salmonids (Chum *Oncorhynchus keta* Walbaum, 1792: 107 ATU, Beacham and Murray, 1987; Coho *Oncorhynchus kisutch* Walbaum, 1792: 120 ATU, Konecki *et al.,* 1995). Thus, genetic differences in development rate between the Exploits River population and the populations used to develop these models could explain their poorer performance. Based on our experiment, the Gorodilov model provided the most accurate predictions, and may be useful for predicting hatch timing in the wild Exploits River population. Atlantic salmon from the north eastern coast of Newfoundland are currently considered as a single evolutionarily unit (COSEWIC, 2010), and range-wide genetic analysis highlights the similar genetic structure of these populations (Jeffery *et al.,* 2018). It would be informative to test whether the Gorodilov model can also be used to provide estimates of hatch and emergence timing in other populations within this region, as that could indicate it works with a large percentage of the remaining viable populations on the island.

An understanding of a population’s reproductive phenology can help managers limit the impact of anthropogenic activities (e.g. flow/release from dams, in-water activities, etc.) on spawning success, and juvenile recruitment. Although the timing of the adult migration into the Exploits River is known (Dempson *et al.,* 2017), the timing of spawning activity, hatch and emergence are not well documented. The little information we have comes from an artificial spawning stream that was built in 1967 in Noel Paul’s Brook (48°37'N, 56°19'W), a tributary of the middle Exploits River (O’Connell *et al.,* 1983). The stocking of fry produced from this artificial stream is largely responsible for the establishment of the current anadromous salmon population above the barrier at Grand Falls. Between 1970 and 1980, spawning in this artificial stream occurred in the last week of October and the first week in November, and hatchlings emerged from the gravel in June (O’Connell *et al.,* 1983).

Although some overlap exists, the emergence period predicted by the Gorodilov model for 2006–18 was consistently earlier than that observed by O’Connell *et al.* (1983) in 1970–80 (see Fig. 5). This discrepancy could be the result of a phenotypic response to different incubation temperatures. For example, we used in-stream water temperatures to predict development, which may not be reflective of the intra-gravel temperatures experienced by incubating embryos/alevins (Acornley, 1999; Hanrahan, 2007; Saltveit and Brabrand, 2013). Alternatively, if water temperatures have warmed over the last ~35 years, this could explain why we predicted emergence to occur substantially earlier in 2006–18. Detailed records of incubation temperatures are not available for the Noel Paul’s Brook spawning channel in the 1970s; however, average daily water temperature during emergence (mean: 12.9°C, range: 11.5–13.8°C, Mercer and Anderson, 1974; Davis and Farwell, 1975; O’Connell *et al.,* 1983) is within the range seen during the emergence windows predicted for 2006–18 (11.3°C; 7.2–16.7°C). Additionally, monthly average water temperatures during incubation from 1977–93 were similar to those in 2006–18, especially in the site located just downstream of the Noel Paul’s Brook tributary (see Supplementary Figure D). Thus, while differences in incubation temperature are likely a contributing factor, temperature alone cannot fully explain the discrepancy in predicted and observed emergence periods.

Technical issues with the Gorodilov model may also influence the accuracy of its predictions. Hatching is a clearly defined and irreversible early life event, whereas emergence from the gravel is a less well defined and potentially reversible event that is governed by behavioural as well as physiological mechanisms (Crisp, 1988; Quinn, 2005). Models were evaluated based on the timing of hatch in our laboratory experiment; since we did not raise alevins to swim-up/emergence, we assumed that the Gorodilov model also accurately predicts development between hatch and emergence. If the Gorodilov model underestimates the ATU required for development between hatch and emergence in the Exploits River population, this would cause us to systematically predict emergence to occur earlier than it actually would. Rearing conditions may also influence the accuracy of model predictions for embryos incubating in the wild. Embryos reared in incubation trays/screens tend to consume the yolk sac faster, and exhibit swim-up behaviour earlier than embryos incubated in artificial/gravel substrates (Crisp, 1988; Beer, 1999; Quinn 2005). The Gorodilov model is based on embryos reared in incubations trays; thus, it may consistently predict earlier emergence than would be seen in alevins incubating within gravel. When possible, we recommend that predictive models be developed using embryos reared under natural thermal regimes and in natural gravel substrate, to avoid these technical issues when the models are used to predict the timing of hatch in wild populations.

Although the Gorodilov model produced the most accurate hatch predictions in our laboratory rearing-experiment, the other three models tested underestimated hatch timing by only 21–26 ATU. This translates to 2–4 days at the temperatures experienced in the wild during the hatching period, which is well within the expected hatching window. Using a combination of all four models to predict hatch timing in the wild may be a more conservative approach given the uncertainty in the date of spawning and spatial variation in environmental conditions experienced by embryos incubating in the wild. A conservative approach is recommended when predicting early life phenology especially considering that climate change is expected to cause water temperatures to increase, and all four models we tested were least accurate when estimating hatch under the warm exposure treatment. Further testing of these models at warm incubation regimes is warranted.

In the wild, spawning within a single population can occur over several weeks (Fleming, 1996). Embryos fertilized late in the spawning period may develop more efficiently than those fertilized early (Hebert *et al.,* 1998; Hendry *et al.,* 1998; Echave *et al.,* 2017; Beer and Steel, 2018), causing hatching to occur within a shorter timeframe than spawning. This is consistent with our results in that embryos fertilized on the two later dates required fewer ATU to hatch in both varying temperature incubation treatments. The slightly warmer temperatures experienced by early fertilized embryos during the first few days of incubation cannot explain the effect of fertilization date on hatch timing because a similar pattern in early thermal environment occurred for embryos incubated at a constant temperature, and there was no effect of fertilization date on hatch timing in this treatment. Accelerated development of late spawned embryos may be related to the relative exposure of fall cooling and spring warming during incubation through either genetic (Hebert *et al.,* 1998) or plastic effects (see the ‘Expansion-compression threshold effect’ proposed by Beer and Steel, 2018). In our study, embryos fertilized on the first two dates spent 4–10 days longer incubating in warmer temperatures prior to exposure to varying thermal conditions compared with embryos fertilized on the latter two dates. Thus, compensatory development associated with the fewer thermal units accumulated in later fertilized embryos could explain why these embryos required fewer ATU at hatch. Our results suggest that even a few days difference in spawn timing may influence ATU required to hatch if incubation temperatures are not constant.

Our modelling exercise predicted substantial variation in the timing and duration of hatch and emergence in Atlantic salmon in the wild. Similar patterns have been identified in Pacific salmon (e.g. Adelfio *et al.,* 2018; Sparks *et al.,* 2018). In our study, site-specific thermal conditions resulted in partially asynchronous hatch and emergence timing among sites within a given year. Such diversity in early life phenology may help buffer overall fry production in the system to variability in climate (Schindler *et al.,* 2010; Adelfio *et al.,* 2018), especially if winter thermal anomalies cause emergence to coincide with unfavourable rearing conditions in some sites. The substantial inter-annual variability in hatch and emergence timing we predicted between 2006–18 suggests that the Exploits River Atlantic salmon are adapted to variable winter climatic conditions. Plasticity in spawn timing and developmental compensation will act to buffer early life phenology of Exploits River Atlantic salmon against changes in incubation conditions; however, whether this plasticity will be sufficient to maintain successful fry production in the face of potentially dramatic changes in winter climates in the future is yet to be seen.

Exposure to different temperatures during incubation may have impacts on development other than hatch timing, which would not have been identified in our study. Incubation temperature can influence size and developmental stage of alevins at hatch (Peterson *et al.,* 1977; Sparks *et al.,* 2017, but also see Steel *et al.,* 2012; Penney *et al.,* 2018). Smaller, less well-developed alevins would be more vulnerable to predators, and fluctuations in prey availability or environmental conditions. Exposure to extreme temperatures during incubation is linked with greater prevalence of morphological deformities in Atlantic salmon (Takle *et al.,* 2005; Wargelius *et al.,* 2005), which would also impact survival. Warmer incubation temperatures can influence juvenile growth trajectories, and increase the proportion of younger smolts (Jonsson *et al.,* 2005; Finstad and Jonsson, 2012). Jonsson *et al.* (2014) found that Atlantic salmon eggs incubated in warm temperatures produced faster-growing juveniles that invested relatively more energy in gonad development as adults. These studies suggest that developmental effects early in life could have important implications for the expression of adult life histories (Clarke *et al.,* 2016), which can affect a population’s sensitivity to harvest (Purchase *et al.,* 2005).

Our study highlights the consistency of hatch timing in response to incubation temperature among Atlantic salmon maternal families from the Exploits River, indicating that changes in winter climate will likely impact the developmental phenology of the entire population in a similar manner. Unless spawn timing responds to climate change in a compensatory way, the ultimate consequence to population dynamics may depend on how the phenology of other environmental factors also respond to changing climates. If warmer winter temperatures advance salmonid development such that peak fry abundance occurs under unfavourable temperatures, or if prey availability does not advance in a similar timeframe, a mismatch between developmental phenology and environmental phenology could be established (Winder and Schindler, 2004; Thackeray *et al.,* 2010), which could lead to reduced juvenile survival. Alternatively, if the phenology of spring environmental conditions and availability of prey advances in a similar manner to that of development, this would result in a prolonged first year growing period, and larger age 1 fish, which could improve juvenile survival. We know that the adult spawning migration in the Exploits River has advanced in response to a changing climate (Dempson *et al.,* 2017). Investigating the inter-annual variation the phenology of fry emergence and prey availability is an important next step in assessing how climate change will likely impact recruitment in wild Atlantic salmon populations.

## Supplementary Material

Winter_climate_and_the_hatch_phenology_of..._coz015
