# Supplementary material for: The impact of a changing winter climate on the hatch phenology of one of North America’s largest Atlantic salmon populations
Source: Conserv Physiol. 2019 May 15;7(1):coz015. doi: 10.1093/conphys/coz015 (PMC6518925; doi:10.1093/conphys/coz015)
Supplement: Winter_climate_and_the_hatch_phenology_of..._coz015 [file winter_climate_and_the_hatch_phenology_of..._coz015.docx]

Supplementary Material

Rooke, Palm-Flawd, Purchase. The impact of a changing winter climate on the hatch phenology of one of North America's largest Atlantic salmon populations.

**Supplementary Material "A"-** Hatch success in laboratory rearing experiment


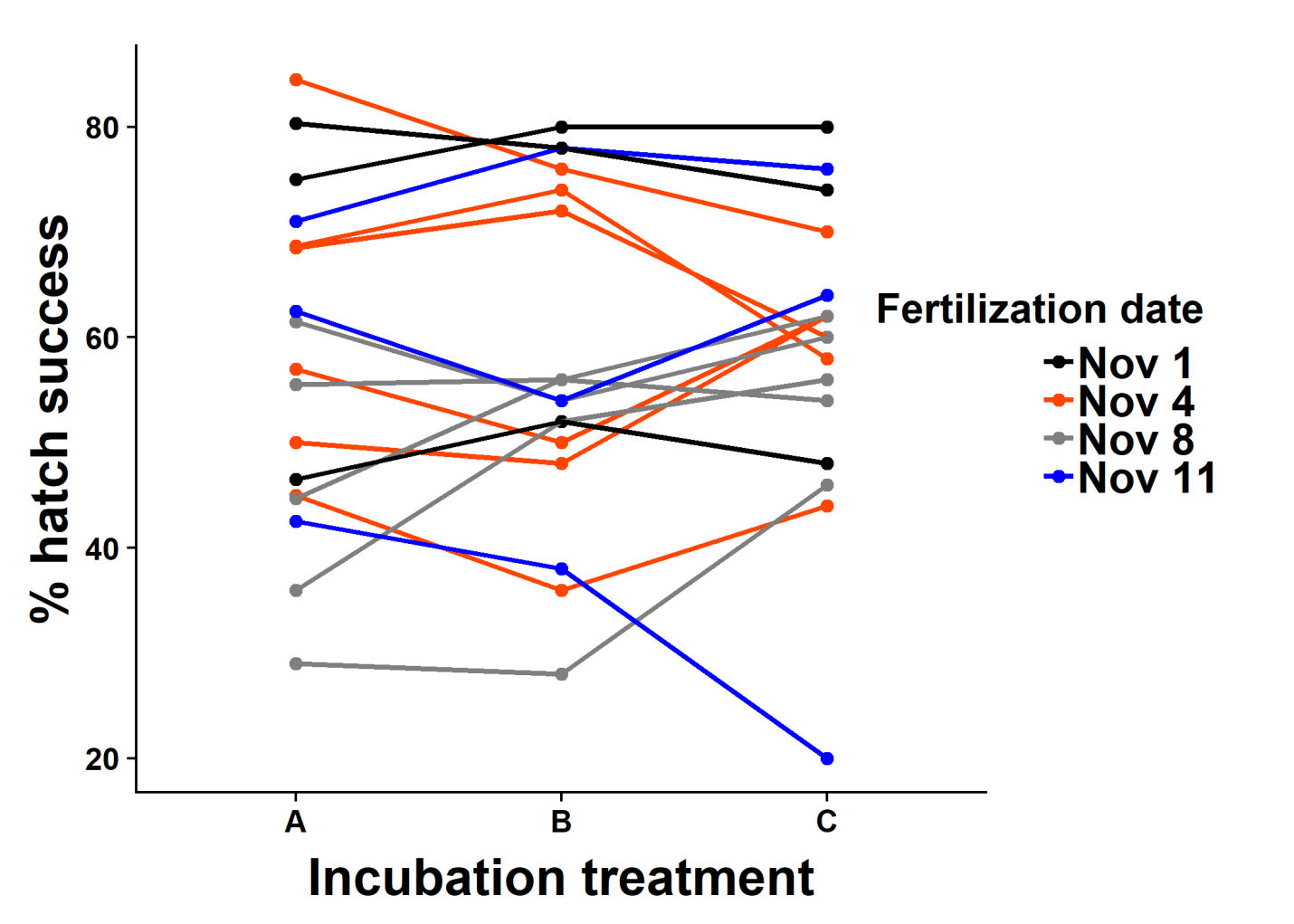


**Figure A:** Percent hatch success for Atlantic salmon embryos from the Exploits River exposed to different incubation temperatures. Each line represents eggs from a single maternal family incubated at constant ~ 5°C (Treatment A), and varying (Treatment B - warm spike, Treatment C - cold spike) thermal conditions. Colors depict embryos fertilized on different dates in November 2016.

**Supplementary Material "B" - Summary statistics for mixed model analysis of hatch timing of Atlantic salmon embryos.**

Table B1 - Summary statistics of mixed model analyses testing the effect of fertilization date on accumulated thermal units (ATU) at 50% and 90% hatch in Exploits River Atlantic salmon embryos. A total of 17 maternal families were tested, with 4−6 replicate incubation tubes reared at a constant 5.3°C. The significance of fixed effect terms was assessed using the Satterthwaite approximation, and the significance of random effects as assessed using likelihood ratio tests.

|  | Fixed Effects | | | | | Random effects | | | |
| --- | --- | --- | --- | --- | --- | --- | --- | --- | --- |
|  | Variable | Mean Sq. | DF | F | P | Variable | Chi Sq. | DF | P |
| ATU at 50% hatch | Fertilization date | 108.3 | 3, 17.5 | 3.67 | 0.032 | Maternal family | 21.78 | 1 | < 0.0001 |
| ATU at 90% hatch | Fertilization date | 112.3 | 3, 17.2 | 2.50 | 0.093 | Maternal family | 31.29 | 1 | <0.0001 |

Table B2 - Summary statistics of mixed model analyses testing the effect of thermal incubation treatment, and fertilization date on accumulated thermal units (ATU) at 50% and 90% hatch in Exploits River Atlantic salmon embryos. A total of 17 maternal families were tested, with a single incubation tube under each of three thermal incubation treatments. The significance of fixed effect terms was assessed using the Satterthwaite approximation, and the significance of random effects as assessed using likelihood ratio tests.

|  | Fixed Effects | | | | | Random effects | | | |
| --- | --- | --- | --- | --- | --- | --- | --- | --- | --- |
|  | Variable | Mean Sq. | DF | F | P | Variable | Chi Sq. | DF | P |
| ATU at 50% hatch | Treatment | 9317.8 | 2, 26 | 569.8 | <0.0001 | Maternal family | 8.04 | 1 | 0.0046 |
|  | Fertilization date | 209.8 | 3, 13 | 13.0 | 0.0003 |  |  |  |  |
|  | Treatment *X* Fertilization date | 313.0 | 6, 26 | 19.1 | <0.0001 |  |  |  |  |
|  |  |  |  |  |  |  |  |  |  |
| ATU at 90% hatch | Treatment | 9772.1 | 2, 26 | 198.3 | <0.0001 | Maternal family | 5.36 | 1 | 0.021 |
|  | Fertilization date | 518.8 | 3, 13 | 10.5 | 0.0009 |  |  |  |  |
|  | Treatment *X* Fertilization date | 451.2 | 6, 26 | 9.2 | <0.0001 |  |  |  |  |

**Supplementary Material "C"-** Statistical analysis of observed accumulated thermal units (ATU) at 90% hatch in laboratory rearing experiment

Table C: Mean + SD incubation temperature (°C) from day of fertilization until the last embryo hatched, accumulated thermal units at 90% hatch (ATU_90_), and days post fertilization at 90% hatch (DPF_90_) for each treatment and fertilization date.

| Fertilization date | Treatment A (constant) | | | Treatment B (warm spike) | | | Treatment C (cold spike) | | |
| --- | --- | --- | --- | --- | --- | --- | --- | --- | --- |
|  | °C | ATU_90_ | DPF_90_ | °C | ATU_90_ | DPF_90_ | °C | ATU_90_ | DPF_90_ |
| Nov 1 | 5.3 + 0.38 | 542 + 4.4 | 102 + 0.8 | 5.2 + 1.92 | 564 + 10.1 | 109 + 2.9 | 3.9 + 1.40 | 504 + 2.7 | 127 + 0.8 |
| Nov 4 | 5.3 + 0.24 | 546 + 14.1 | 104 + 2.8 | 5.2 + 1.90 | 545 + 10.5 | 107 + 3.1 | 3.9 + 1.33 | 496 + 6.8 | 128 + 2.0 |
| Nov 8 | 5.2 + 0.15 | 544 + 3.9 | 104 + 0.8 | 5.1 + 1.91 | 530 + 9.8 | 107 + 2.9 | 3.8 + 1.25 | 489 + 10.1 | 129 + 2.9 |
| Nov 11 | 5.2 + 0.15 | 531 + 1.7 | 102 + 0.3 | 5.2 + 1.94 | 506 + 2.5 | 102 + 0.8 | 3.7 + 1.24 | 491 + 3.2 | 132 + 0.9 |
| Treatment total | 5.3 + 0.38 | 542 + 10.0 | 103 + 2.0 | 5.2 + 1.92 | 537 + 20.1 | 106 + 3.3 | 3.9 + 1.37 | 495 + 8.6 | 129 + 2.4 |

When incubated at a constant 5°C, the ATU required for 90% hatch was considered moderately repeatable among replicate incubation tubes within a maternal family (ICC = 0.67; 95%CI: 0.48−0.84). Although hatch timing was significantly different among maternal families (*χ^2^_1_* = 31.3, *P* <0.0001; Figure C1), the maximum difference between two families (34 ATU, F5 vs. F9, Figure B1) was small relative to the average total ATU at hatch across all maternal families (541 ATU). Thus, the among maternal family variability in hatch timing represented only 6.3% of the average total incubation period. ATU at 90% hatch was not significantly different among fertilization dates (F_3,17.2_ = 2.50, *P* = 0.09). Considered together, intra- and inter- maternal family variability in hatch timing was small relative to the average total duration of incubation, indicating that the timing of hatch was similar among maternal families.


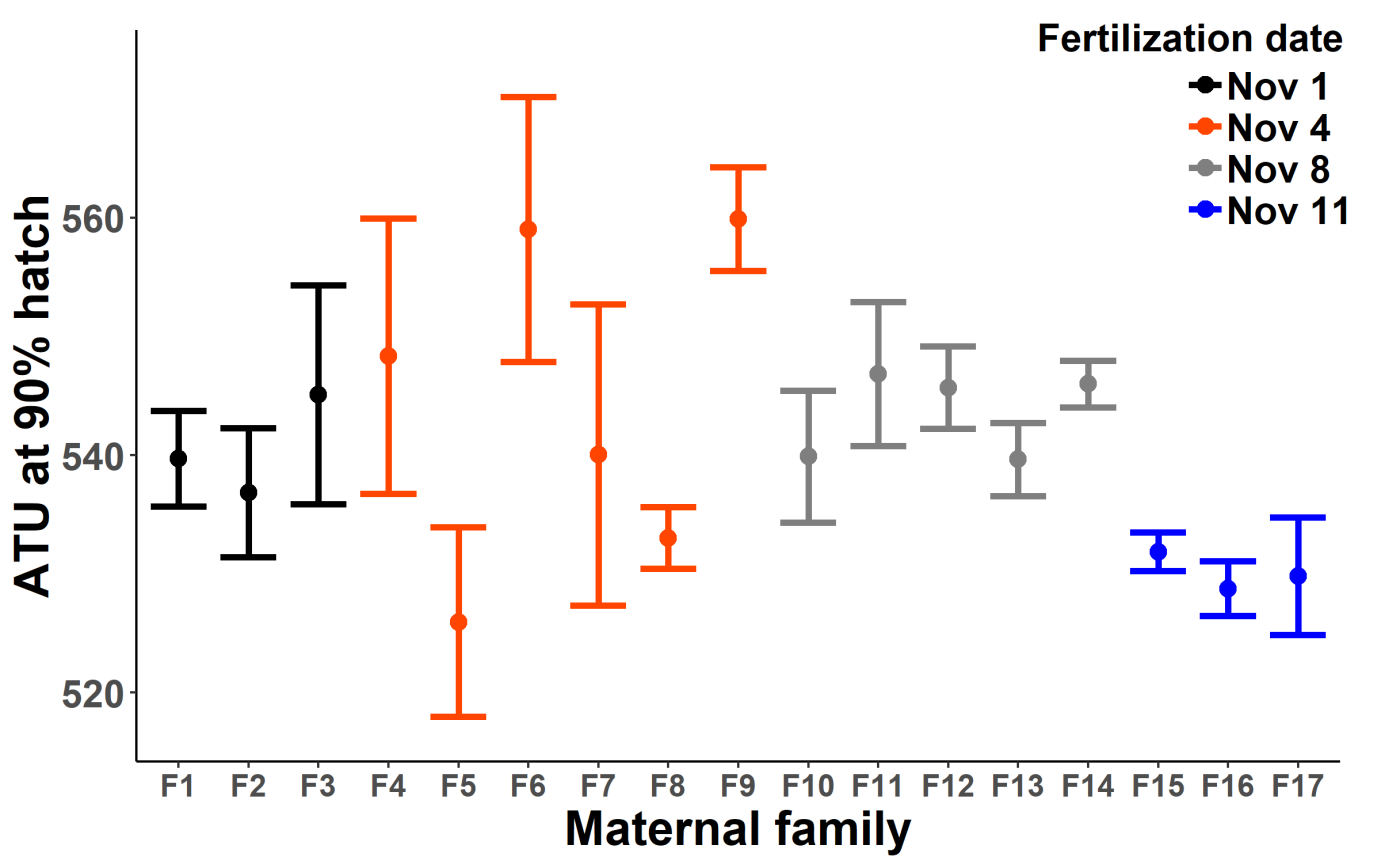


**Figure C1:** Intra- and inter-maternal family variability in accumulated thermal units (ATU) post fertilization at 90% hatch of Atlantic salmon embryos from the Exploits River incubated at constant ~5.3°C (Treatment A). Points show average + SD of 4−6 replicate incubation tubes per maternal family. Colors depict embryos fertilized on different dates in November 2016.

**
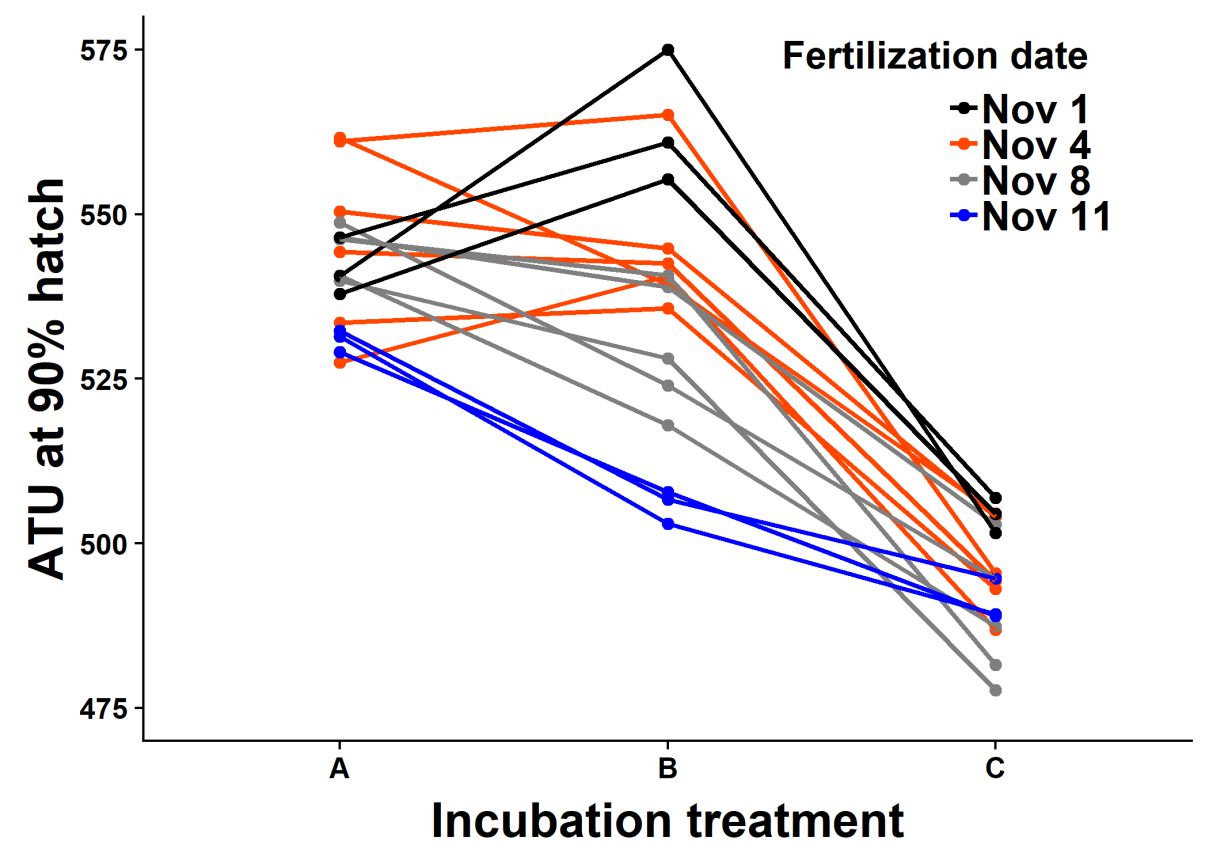
**

**Figure C2:** Accumulated thermal units (ATU) post fertilization at 90% hatch for embryos from Atlantic salmon from the Exploits River exposed to different incubation temperatures. Each line represents embryos from a single maternal family incubated at constant (Treatment A: mean + SD = 5.3 + 0.38°C), and varying (Treatment B: 5.2 + 1.92°C - warm exposure, Treatment C: 3.9 + 1.37°C - cool exposure) thermal conditions.

A significant interaction between incubation treatment and date of fertilization (F_6,26_ = 9.16, *P* < 0.0001) precluded a statistical evaluation of the effect of temperature on hatch timing; however, the response to temperature is clear from Figure B2: embryos incubated at constant 5.2°C, and those exposed a warm spike, had similar average ATU at hatch, while those exposed to a cold spike required fewer ATU to hatch. Although the timing of hatch differed a little among families, the effect of temperature was consistent across maternal families (i.e. a maternal family that required relatively few ATU at hatch in one incubation treatment also tended to require relatively few ATU at hatch in another treatment, χ^2^_1_ = 5.36, *P* = 0.02).

When briefly exposed to warm temperatures, and to a lesser degree when exposed to cold temperatures, maternal families fertilized on November 8^th^ and 11^th^ tended to require fewer ATU at 90% hatch, compared to families fertilized on November 1^st^ or 4^th^. However, the largest difference in ATU between fertilization dates (72 ATU, Nov 1^st^ vs. Nov 11^th^ in Treatment B, Figure C2) was small compared with the average total ATU at hatch in the treatment (537 ATU). Thus, the effect of fertilization date on hatch timing was relatively small, representing at most only 13.4% of the average duration of the entire incubation period within a treatment.

**Supplementary Material "D"** - Comparison of water temperatures in the Exploits River during the incubation period from 1977−1993, and 2006−2018.


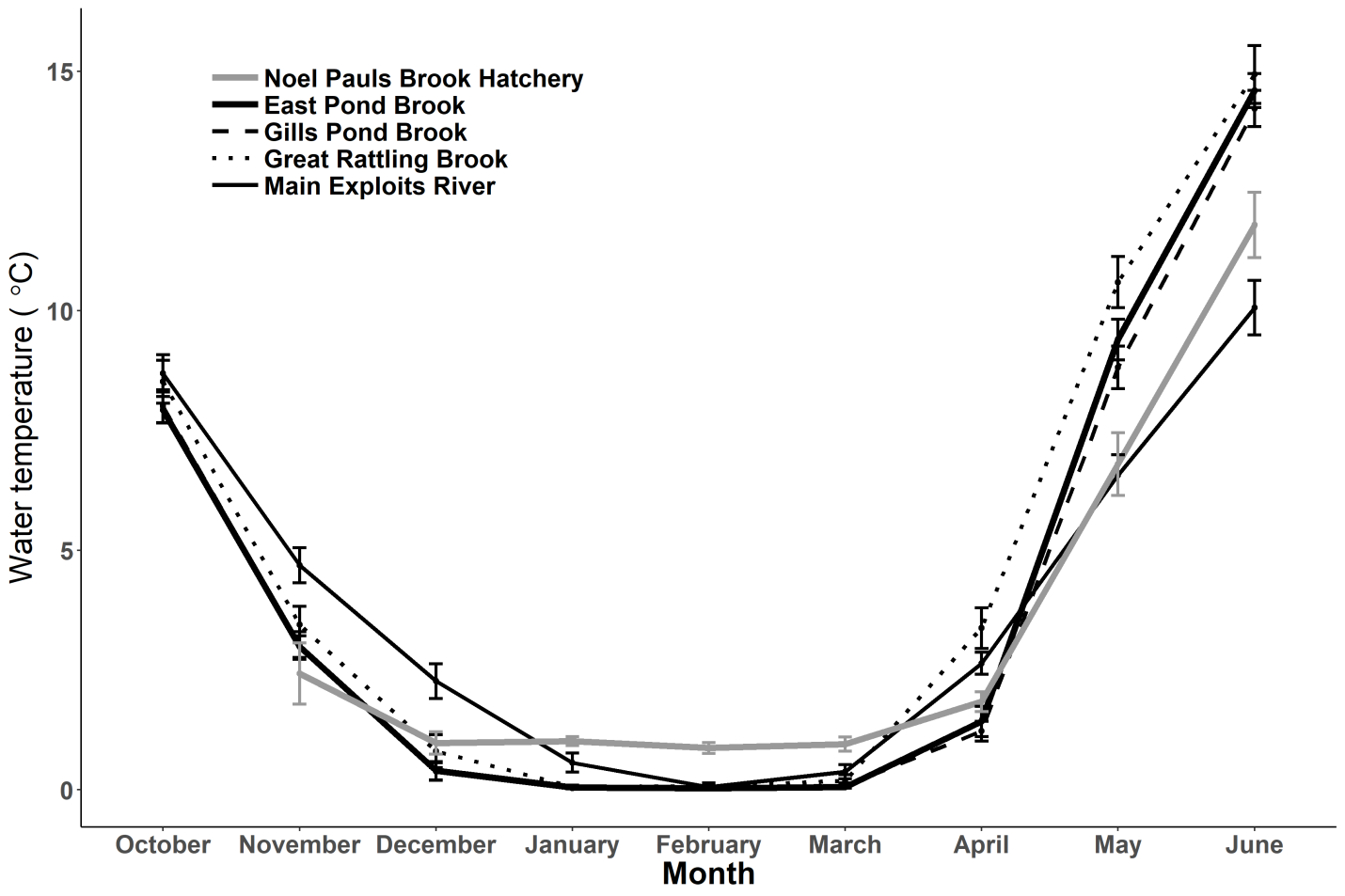


**Figure D:** Monthly average water temperature (°C) in the Exploits River in 1977−1993 (grey), and 2006−2018 (black - line types depict different locations within the system). Error bars show + SE of yearly averages. Data source: Department of Fisheries and Oceans (1977−1993) Annual summary of the Exploits River Atlantic salmon development program, Unpublished reports, Government of Canada; Water Resources Department (2006−2018) Government of Newfoundland and Labrador, https://www.mae.gov.nl.ca/wrmd/ADRS/v6/Graphs_List.asp.
